# pH and Glucose Dual-Responsive Hybrid Polymeric Smart Insulin Carrier for Diabetes Treatment

**DOI:** 10.3390/polym18101209

**Published:** 2026-05-15

**Authors:** Kyu Oh Kim

**Affiliations:** Department of Fiber System Engineering, Dankook University, 152, Jookjeon-ro, Suji-gu, Yongin-si 448-701, Gyeonggi-do, Republic of Korea; kokim95@dankook.ac.kr; Tel.: +82-31-8005-3561

**Keywords:** glucose-responsive insulin delivery, pH-sensitive nanoparticles, POSS-based drug carrier, phenylboronic acid (APBA), poly(acrylic acid) (PAA), stimuli-responsive drug delivery, biocompatible nanocarriers, diabetes treatment

## Abstract

Glucose-responsive smart insulin delivery systems that mimic the pancreatic insulin release system can improve the health and quality of life of patients with diabetes. In this study, a spherical drug delivery carrier encapsulating insulin was developed to achieve improved glucose accessibility and a rapid pH response using polyhedral oligomeric silsesquioxane (POSS) as a sterically stabilizing structure. Highly sensitive poly(acrylic acid) (PAA)-POSS-aminophenylboronic acid (APBA)@insulin (386 ± 69 nm), a spherical drug delivery carrier encapsulating insulin, was synthesized using POSS, a hydrophobic material, and PAA and APBA, which respond to pH and glucose, respectively. The drug carrier has dual reactivity with pH and glucose, and the synthesis of the carrier was confirmed through Fourier transform infrared (FT-IR) spectroscopy, which verified that the particles were stable at each pH through the zeta-potential data. In particular, PAA-POSS-APBA@insulin exhibited highly sensitive drug delivery characteristics, in which the backbone of PAA was expanded under acidic conditions (around pH 5.0) and insulin bound to the boronic acid inside could rapidly and selectively react with trace amounts of glucose. PAA-POSS-APBA@insulin nanoparticles exhibited no HeLa cell cytotoxicity up to a high concentration of 640 μg/mL, and the cell growth rate increased with the concentration, indicating biocompatibility. The average blood glucose level of diabetic mice treated with POSS-APBA@insulin (4.0 IU/kg) decreased for >6 h and remained stable. Thus, PAA-POSS-APBA@insulin can function as a stimulatory-responsive drug carrier targeting hyperglycemic environments.

## 1. Introduction

Diabetes is a metabolic disease in which blood glucose (BG) levels increase because of insufficient secretion or abnormal insulin function. According to the International Diabetes Federation (IDF), the number of diabetic adults worldwide reached 537 million in 2021, showing a remarkable increase of 74 million in comparison to 2019 [1,2,3]. Insulin—a hormone that helps cells absorb glucose for energy—is essential for the treatment of type 1 and advanced type 2 diabetes, as it maintains normal blood sugar levels [4,5,6]. However, in contrast to the body’s natural, regulated secretion of insulin, conventional exogenous insulin therapy relies on periodic injections administered by the patient. Insufficient blood sugar control can cause side effects such as limb amputation, blindness, and renal failure, and excessive insulin administration can lead to hypoglycemia, which can cause behavioral and cognitive disorders, seizures, loss of consciousness, brain damage, and even death; therefore, insulin-mimetic technology is needed [7,8,9,10,11,12,13,14,15,16,17].

A “smart” glucose-responsive insulin delivery system that mimics β-cells to release insulin in response to high blood sugar levels is desirable for controlling blood sugar levels with minimal effort and improving the health and quality of life of diabetic patients by regulating homeostasis to maintain optimal glucose concentrations. Insulin smart drugs are drugs that have a switch that turns their activity on and off in response to blood sugar glucose levels. They comprise polymeric biomaterials along with a sensor that detects blood glucose (BG) and an infusion pump that releases insulin to maintain glucose concentrations within a normal range [17,18,19]. Recently, various stimuli-responsive delivery systems based on advanced polymeric biomaterials have been developed to achieve more precise and stable glucose control [20,21]. Enzymes such as glucose oxidase (GOx) are used as glucose-sensing substances, but they have the problem of being easily oxidized during manufacturing. Alternatively, synthetic phenylboronic acid (PBA) has been explored; however, its clinical utility is often limited by the hydrophobic aggregation of phenyl groups. In our previous studies, we addressed this by synthesizing polyhedral oligomeric silsesquioxane–aminophenylboronic acid (POSS-APBA), utilizing the 3D stereostructure of POSS to prevent aggregation and provide sufficient space for glucose interaction [22,23].

Building on this, a smart drug formulation that maintains blood sugar homeostasis required additional regulatory mechanisms; thus, a pH-responsive poly(acrylic acid) (PAA) polymer was selected [24]. Local pH variations often correlate with insulin deficiency; specifically, an acidic microenvironment can be associated with rising glucose levels and metabolic shifts [12]. By employing a pH-responsive outer shell, the system is designed to facilitate the glucose–PBA reaction primarily under specific environmental conditions.

Despite the potential of glucose-responsive systems, achieving rapid-on/off kinetics remains a significant challenge. Traditional GOx-based strategies often suffer from delayed response times and enzyme instability during long-term physiological integration [4]. Recently, Yang et al. demonstrated a glucose-responsive system based on dynamic covalent bonding between PBA and poly(vinyl alcohol) [25]. While such systems exhibit excellent self-healing properties, achieving rapid-on/off kinetics remains a challenge due to the inherent structural density of linear polymer networks, which can delay glucose accessibility. To address these limitations, we have reported a novel 3D hybrid network utilizing polyhedral oligomeric silsesquioxane (POSS) as a structural core.

Unlike conventional linear polymer systems, the POSS framework provides a well-defined nanostructure that suppresses phenylboronic acid (PBA) aggregation and enhances the accessibility of glucose-binding sites, thereby facilitating more efficient glucose diffusion and interaction. Furthermore, the incorporation of a pH-responsive poly(acrylic acid) (PAA) shell introduces a dual-stimuli gating mechanism, enabling the system to respond synergistically to both glucose concentration and local pH variations. This design enables controlled modulation of insulin release in response to physiologically relevant environmental changes. The integration of a rigid inorganic nanocore with a stimuli-responsive polymer shell establishes a tunable network architecture, which influences molecular transport pathways and response kinetics.

In this study, we systematically investigate the relationship between the network structure, intermolecular interactions, and glucose-triggered insulin release behavior. Comprehensive physicochemical characterization, together with in vivo evaluation in diabetic animal models, was conducted to validate the functionality of the system. These results indicate that improved responsiveness and glucose regulation behavior can be attributed to the synergistic effect of the 3D POSS core and the pH-responsive PAA shell.

## 2. Materials and Methods

### 2.1. Materials

PSS-[2-(3,4-epoxycyclohexyl)ethyl]-heptaisobutyl-substituted (POSS), PSS-(2,3-propanediol)propoxy-heptaisobutyl-substituted(POSS), 3-aminophenylboronic acid monohydrate (APBA), human insulin (empirical formula: C_257_H_383_N_65_O_77_S_6_, molecular weight (Mw): 5807.57 g/mol), sodium cyanoborohydride, polyethylene glycol (Mw = 200 g/mol, PEG), poly(acrylic acid) (PAA, Mw: 5000 g/mol partial sodium), sulfuric acid (95.0%), tetrahydrofuran (THF), a hydrazine monohydrate catalyst, and an ammonia solution were obtained from Sigma–Aldrich (Seoul, Republic of Korea) and used as received. All the other chemicals were of analytical grade.

### 2.2. Synthesis of POSS-APBA

For the synthesis of POSS-APBA, 10.00 mg of POSS (epoxycyclohexyl) and 1.67 mg of APBA were added to 9 mL of THF and stirred in an oil bath at 50 °C for 3 h with a mechanical stirrer. Subsequently, 50 μL of hydrazine monohydrate catalyst and 50 μL of ammonia solution were added, stirred for 1 h, and then cooled to room temperature. THF was removed from the synthesized solution in an oven maintained at 45 °C to obtain POSS-APBA powder. The unreacted materials were removed using acetone and washed with water to prepare pure POSS-APBA powder [22].

### 2.3. Synthesis of PAA-POSS

To prepare PAA-POSS, 10.00 g of PAA, 95.0 mg of POSS(2,3-propanediol) and 56 μL of sulfuric acid were dissolved in 50 mL of THF in a 3-neck reactor and stirred under reflux at 100 °C for 5 h with a mechanical stirrer. To remove POSS that did not participate in the synthesis, the synthesized POSS-PAA was filtered with chloroform using a vacuum pump. The filtered compound was dried in a vacuum oven at 150 °C for 30 min to obtain white powder [24].

### 2.4. Synthesis of Modified Insulin

To produce PEG-insulin, 8.0 mg of insulin, 2.00 g of PEG, and 2.0 mg of sodium cyanoborohydride were dissolved in 10 mL of THF and stirred at room temperature for 1 h. After the synthesis was complete, the PEG-insulin was incubated at room temperature for 1 h for stabilization.

### 2.5. Preparation of PAA-POSS-APBA@Insulin

The PAA-POSS-APBA@insulin was prepared via two steps: synthesis and solvent exchange. In the synthesis step, 11.0 mg of the prepared POSS-APBA, POSS-PAA powder, POSS-APBA: POSS-PAA (2:1 wt% ratio), and PEG-insulin DMSO solution were stirred for 2 h and then stabilized for 0.5 h. The stabilized solution in dimethyl sulfoxide (DMSO) was changed to an aqueous solution via dialysis during the solvent-exchange step. The dialysis tubes used were selected according to their pore size to ensure that the PAA-POSS-APBA@insulin nanoparticles (NPs) could not pass through, allowing only the solvent to be exchanged. The stabilized solution was placed in a dialysis tube with a molecular weight cutoff (MWCO) size of 100–500 Da, and the tube was immersed in a water bath filled with distilled water to remove the organic solvent. To minimize the denaturation of insulin due to temperature and light, we performed dialysis in a refrigerator at 3 °C, blocking out light with aluminum foil. The mixture was then poured into a dialysis tube (Spectra/Por 6, MWCO: 6 kDa) and dialyzed against distilled water under magnetic stirring for 1 d to remove all residues. Distilled water in the bath was replaced with fresh distilled water at 0.25, 0.5, 0.75, 1, 3, 6, 12, and 24 h to increase the dialysis efficiency. After dialysis, the PAA-POSS-APBA@insulin solution was stored in a refrigerator at 3 °C. The stability of insulin during the PEGylation and PAA-POSS-APBA encapsulation was previously verified via circular dichroism (CD) spectroscopy, showing no significant conformational changes [23].

### 2.6. Characterization

#### 2.6.1. Fourier Transform Infrared (FT-IR) Spectroscopy

FT-IR spectral analysis was performed using a PerkinElmer Spectrum II spectrometer (Waltham, MA, USA) to confirm the synthesis and structure of the sample. FTIR spectra were obtained from KBr pellets. These pellets were prepared by mixing KBr powder and the sample at a weight ratio of approximately 100:1 using agate induction so that they were well dispersed and then applying pressure using a pellet-molding apparatus. The sample was analyzed in the range of 4000–400 cm^−1^ to confirm the characteristic peaks of POSS-APBA and the changes in peak positions and shapes.

#### 2.6.2. Proton Nuclear Magnetic Resonance (^1^H-NMR)

^1^H-NMR analysis was performed to analyze the structure of the synthesized material. To completely remove the solvent from the POSS-APBA and PAA-POSS-APBA@insulin samples, they were dried in a vacuum oven at 90 °C for 24 h. The samples were then dissolved in deuterated chloroform at a concentration of 0.02 g/0.7 mL and analyzed.

#### 2.6.3. Thermogravimetric Analysis (TGA)

Thermal stability analysis of the synthesized PAA-POSS-APBA@insulin was performed using a TGA N-1000 (Seoul, Republic of Korea). The samples were heated from 20 to 700 °C at a rate of 10 °C/min.

#### 2.6.4. Scanning Electron Microscopy (SEM) and Transmission Electron Microscopy (TEM)

The 3D structures and shapes of the particles were examined using SEM (S-5200) (Hitachi, Tokyo, Japan) and TEM (JEM-1011) (JEOL, Tokyo, Japan). The samples were prepared by dropping POSS-APBA, POSS-APBA@insulin, and PAA-POSS-APBA@insulin NPs stored in distilled water on carbon tape; drying the tape in a vacuum oven at 80 °C for 24 h; and then coating the carbon tape for SEM observation. TEM images were obtained using a JEM-1011 (Philips CM200) transmission electron microscope (Tokyo, Japan) operating at 40–100 kV.

#### 2.6.5. Zeta Potential (ζ)

The zeta potential was measured to determine the variation in the stability of the PAA-POSS-APBA@insulin at different pH values. Zeta-potential measurements were performed using an ELSZ-2000S (Otsuka, Japan) instrument with the samples dispersed in distilled water, where the pH was controlled with 0.1 N HCl and 0.1 N NaOH. The zeta potential is difficult to measure directly, and the typical route is to determine it indirectly by measuring the electrophoretic mobility (particle velocity divided by the electric-field strength) under an applied electric field according to Henry’s equation (Equation (1)):

(1) $$\frac{U}{E}=\frac{2\epsilon \zeta F(\kappa \alpha )}{3\eta }$$

where *U*/*E* represents the electrophoretic mobility (m^2^ s^−1^ V^−1^), *ζ* represents the zeta potential (V), *ε* represents the solvent dielectric permittivity (or constant) (kg m V^−2^ s^−2^), *η* represents the viscosity (kg m^−1^ s^−1^), and *κa* represents the ratio of the particle size to the Debye length (1/*κ*). Thus, *κa* ≫ 1 indicates that the particle radius (*a*) is large compared to 1/*κ* (1/*κ* is ∼10 nm for 1 mM aqueous salt solutions) [26].

#### 2.6.6. Zeta Sizer

A Zetasizer Nano ZS90 (Malvern Instruments, Worcestershire, UK) was used to measure the particle size and behavior of POSS-APBA and POSS-APBA@insulin under various pH conditions and sugar concentrations. The zeta size was measured using a Zetasizer Nano ZS90 (Malvern Instruments, Worcestershire, UK), with each sample dispersed in distilled water. To measure the zeta size, hydrochloric acid and sodium hydroxide were added to the distilled-water solution to adjust the pH to 2, 4, 6, 7, 8, and 10. The measurements were performed at low, medium, and high glucose concentrations.

#### 2.6.7. In Vitro Drug Loading Efficiency

To determine the loading efficiency of insulin in POSS-APBA NPs, the amount of free insulin in supernatants was assayed using UV–vis spectrophotometry. The drug loading efficiency was calculated using the equations below. Insulin entrapment efficiency (EE) and loading capacity (LC) were calculated using the following equations:

(2) $$Insulin\;entrapment\;efficiency\;(EE\%)=\frac{Total\;Insulin-Free\;Insulin}{Total\;Insulin}\times 100$$

(3) $$Insulin\;loading\;capacity\;(LC\%)=\frac{Total\;Insulin-Free\;Insulin}{Nanoparticles\;weight}\times 100$$

#### 2.6.8. Cell Lines and Culture Condition

HeLa and human dermal fibroblast (HDF) cells were purchased from the American Type Culture Collection (ATCC) and maintained in DMEM supplemented with 10% fetal bovine serum (FBS) and 1% penicillin/streptomycin. Human umbilical vein endothelial (HUVE) cells were kindly provided by Professor Chung-Hyun Cho (Seoul National University College of Medicine, Republic of Korea) and maintained in M199 medium supplemented with 10% FBS and 1% penicillin/streptomycin. These cells were maintained in a humidified atmosphere containing 5% CO_2_ at 37 °C.

#### 2.6.9. Cell Viability Assay

Cells were seeded in 96-well culture plates and incubated in the culture medium until they reached 80% confluence. The cells were treated with various concentrations of the samples for 24 h and incubated with the MTT reagent for 2 h. Blue formazan crystals were solubilized in DMSO, and the formazan levels were determined at 570 nm using an Infinite M200 PRO plate reader (Tecan Group Ltd., Männedorf, Switzerland). All experiments were performed in quintuplicate (*n* = 5). Data are presented as mean ± standard deviation (*n* = 3).

#### 2.6.10. In Vivo Studies Using Streptozotocin (STZ)-Induced Diabetic Mice

All procedures were performed under an animal protocol approved by the Institutional Animal Care and Use Committee of the Biomedical Research Institute at the Seoul National University Hospital (IACUC No. 13–0277-C3A2; approved on 7 February 2013). The study protocol adhered to relevant ethical guidelines and regulations. Female C57BL/6 mice were rendered diabetic via daily intraperitoneal injection of STZ dissolved in 10 mM citrate buffer (pH 4.5) at a dose of 75 mg/kg body weight for 3 d [23]. Mice were considered diabetic when their fasting BG levels were >600 mg/dL one week after STZ treatment [24]. The hypoglycemic activity of the PAA-POSS-APBA@insulin NPs was evaluated via subcutaneous administration of the test NPs (4.0 IU/kg) in diabetic mice fasted overnight. Untreated mice and those injected with PAA-POSS-APBA@insulin NP solution (4.0 IU/kg) were used as controls (*n* = 4 for each studied group). Blood samples were collected from the tail vein of the rats prior to drug administration and at different time intervals after dosing. The BG levels were immediately determined using a glucose meter (Accu-chek^®^ Inform II glucose meter, Roche, Burgess Hill, UK).

#### 2.6.11. Statistical Analysis

Comparisons between groups were performed using the one-tailed Student’s *t*-test (SPSS, Chicago, IL, USA). All data are presented as the mean ± standard deviation. Differences were considered statistically significant when *p*-values were <0.05.

## 3. Results and Discussion

**Figure 1:**
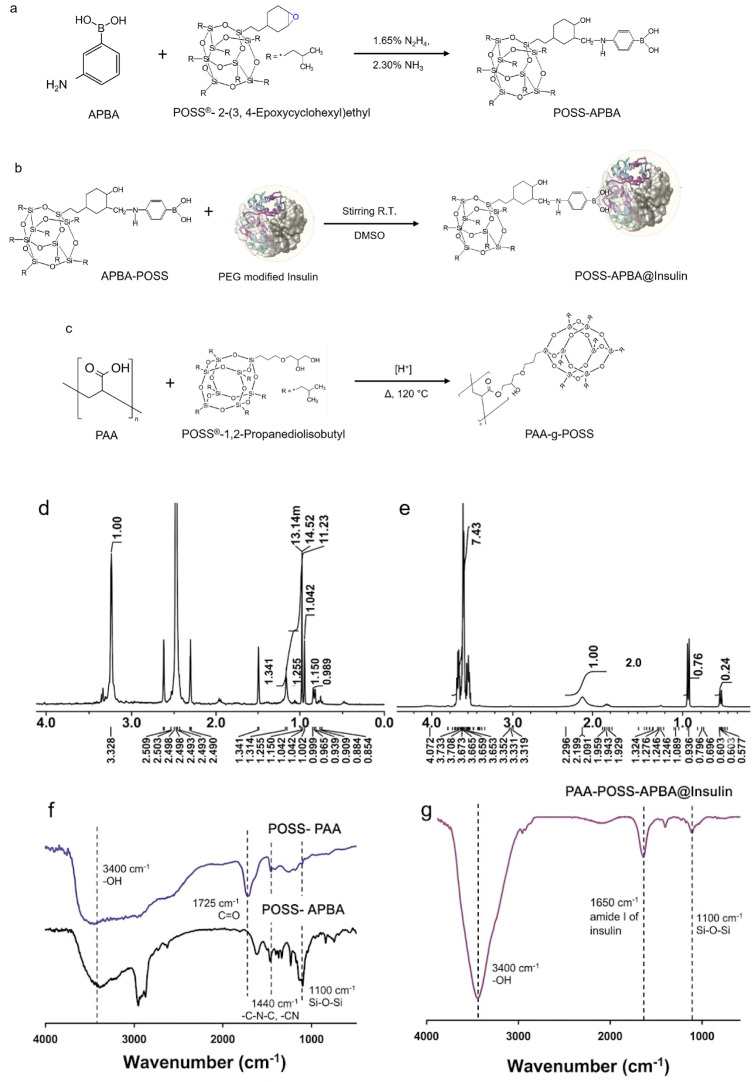
Synthesis of (**a**) APBA-POSS, (**b**) POSS-APBA@insulin carrier [EE: 75.6%, LC: 51.0%], and (**c**) PAA-POSS; ^1^H-NMR spectra of (**d**) POSS-APBA and (**e**) PAA-POSS-APBA@insulin; FT-IR spectra of (**f**) POSS-APBA, POSS PAA, and (**g**) PAA-POSS-APBA@insulin. (*: denotes the attachment point to the polymer backbone).

The smart response of the PAA-POSS-APBA@insulin system relies on the reversible competitive binding between glucose and the hydroxyl groups of modified insulin toward the APBA moiety. This on/off switching mechanism was established in our previous studies, where the carrier effectively regulated insulin release in response to fluctuating glucose levels. As shown in Figure 1, an organic synthetic probe that reacts with BG was fabricated by synthesizing APBA using POSS. In a previous study, we drew a parallel between the response rates of POSS–APBA–dye and APBA–dye probes for various concentrations of glucose ranging from 0 to 5 mg/mL [22]. In the absence of POSS, the response was unaffected by increasing glucose concentrations; however, the response of the probe containing POSS changed. The aromatic hydrocarbons in APBA form tightly packed crystals, as indicated by a higher packing coefficient, owing to the large surface area of intermolecular contact and van der Waals interactions [27,28]. Therefore, by synthesizing compounds containing aromatic rings, such as benzene, with the 3D cubic structure of POSS, d-glucose provides a space in which boronic acid can easily approach the surrounding d-glucose. It is a highly sensitive sensor probe that can react in high yields even in trace amounts. Additionally, our previous study demonstrated that amphiphilic POSS-PEG–PEG and POSS-PVA telechelics aggregate into flower-shaped micelles with hydrophilic groups facing outward owing to the strong hydrophobic interaction of the POSS moiety in an aqueous environment [29,30]. This is a smart drug delivery system in which PEG-modified insulin is loaded onto boronic acid through hydrogen bonding and electrostatic forces and insulin is supplied through an exchange reaction with nearby d-glucose. The synthetic process is shown in Figure 1b, where the drug release of the synthesized POSS-APBA@insulin and the reactivity between glucose concentrations are linearly proportional (y = 185.83x + 30.89 and R^2^ = 0.9792), indicating sensor-like accuracy and high reactivity [23]. However, although an appropriate amount of glucose is required in the blood, the proposed POSS-APBA@insulin system may cause hypoglycemia. Therefore, when a diabetic patient has a shortage of insulin, his/her blood sugar level rises, and because glucose cannot be used as an energy source, the inner mitochondrial uncoupling protein induces superoxide generation and further hyperpolarizes the mitochondrial membrane, creating an environment that stimulates oxidative stress [26,31,32,33]. Thus, we used a PAA polymer, whose interior particle structure opens when the environment becomes acidic. In addition, we synthesized PAA-POSS with high reactivity and structured it to react sensitively with an acidic environment and suppress the denaturation of insulin drugs (Figure 1c) [24]. All compounds were synthesized using a mild method without toxic additives or catalysts, and their biocompatibility was evaluated to confirm that they were suitable for use as drugs.

First, POSS-APBA@insulin was synthesized, and then the POSS-PAA composite was added to a dialysis tube at a weight ratio of 2:1 to obtain PAA-POSS-APBA@insulin. Figure 1d shows the H-NMR data of POSS-APBA and PAA-POSS-APBA@insulin and the FT-IR spectra of POSS-APBA, POSS PAA, and PAA-POSS-APBA@insulin, confirming that the synthesis was successful. The characteristic peaks of PAA can be observed, along with those of POSS at 1.85 (^7^H, -Si-CH_2_CH(CH_3_)_2_), 1.43 (^14^H, -Si-CH_2_CH(CH_3_)_2_), and 0.95 (^42^H, -Si-CH_2_CH(CH_3_)_2_).

The peaks at 1000–1300 cm^−1^ are due to the Si–O–Si stretching vibrations of POSS, confirming the integrity of the cage structure. The C–N stretching peak of APBA and the POSS peak at 1240 cm^−1^ are due to the C–O stretching vibrations of the epoxide group [22]. The new peak at 1470 cm^−1^ in the POSS–APBA spectrum is due to C–N stretching, second amine C–N–C stretching, C–C stretching (phenyl ring), and B–O stretching of APBA, but these peaks are absent in the spectra of POSS. The band at 3350 cm^−1^ can be assigned to the N-H stretching of the secondary amine in POSS-APBA. Peaks corresponding to ester groups of PAA-POSS can be observed at 1715 and 1735 cm^−1^. The band at ~1650 cm^−1^ is attributed to the amide I vibration of insulin, with possible partial overlap from the carbonyl groups of the polymer matrix. The broadening and slight shift in the carbonyl and amide bands suggest the formation of hydrogen bonding interactions between the carboxyl groups of PAA and the amine groups of insulin and POSS-APBA. These interactions are expected to contribute to the stabilization of the hybrid network and influence the diffusion behavior of glucose and insulin.

Furthermore, the preservation of characteristic peaks alongside subtle spectral shifts indicates that the hybrid network is formed through non-covalent interactions rather than structural degradation of the components. This suggests that the designed system maintains structural integrity while enabling dynamic and stimuli-responsive behavior.

The TGA curve of PAA-POSS-APBA@insulin demonstrates enhanced thermal stability compared to its individual components, attributed to the synergistic effect of chemical cross-linking and the hybrid architecture in the Supporting Information (Figure S2). The formation of boronate ester bonds between APBA and insulin effectively suppresses the thermal denaturation of the protein. Furthermore, the inorganic POSS framework uniformly dispersed within the organic matrix acts as a robust thermal barrier, providing structural rigidity. The high residual weight (approximately 25–30%) at 700 °C confirms the presence of the inorganic SiO_2_ core and the stable cross-linked network. These results verify the successful chemical integration of insulin within the 3D hybrid nanocarrier.

**Figure 2:**
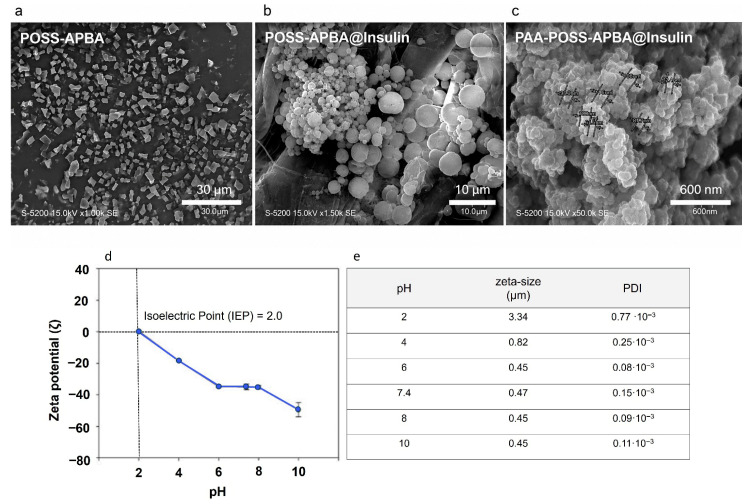
FE-SEM images of (**a**) POSS-APBA, (**b**) POSS-APBA@insulin, and (**c**) PAA-POSS-APBA@insulin; (**d**) zeta potentials and (**e**) sizes of PAA-POSS-APBA@insulin under various pH conditions. Data are presented as mean ± standard deviation (*n* = 3).

Field-emission scanning electron microscopy (FE-SEM) was employed to evaluate the structural evolution of the synthesized platforms (Figure 2a–c). Pristine POSS-APBA exhibited a well-defined octagonal crystalline habit, a morphology attributed to the characteristic weak hydrogen bonding interactions between –OH and –NH moieties. Upon the encapsulation of insulin, a distinct transition to smooth, spherical micellar architectures was observed for POSS-APBA@insulin. This morphological shift suggests that the primary hydrogen-bonding network of the POSS framework is disrupted in favor of covalent or coordinative interactions between the boronic acid groups and PEG-modified insulin, driving the self-assembly of spherical micelles. The hierarchical assembly of PAA-POSS and POSS-APBA@insulin resulted in a complex, “grape-like” morphology (Figure 2c). Quantitative analysis of these clusters revealed a primary subunit size of DM_ave_ = 64.95 ± 24.03 nm. Transmission electron microscopy (TEM; see Figure S1) further confirmed that these subunits aggregated into larger stable assemblies with a mean diameter of 386 ± 69 nm. Such multi-scale architecture is highly advantageous for reactive particle applications, as the granular surface topology significantly enhances the effective surface area compared to monolithic spheres. Statistically, the transition from bulk POSS-APBA (2.54 ± 0.8 μm) to the insulin-loaded POSS-APBA@insulin (298 ± 121 nm) demonstrates a significant reduction in hydrodynamic dimensions. These size distributions remain within the optimal range for systemic circulation, ensuring hemocompatibility and unobstructed transport through the microvasculature [11]. Figure 2d,e show the zeta-potential and zeta-size data of PAA-POSS-APBA@insulin under various pH conditions. At all the pH values, PAA-POSS-APBA@insulin had a negative charge, and as the pH changed to neutral and alkaline, it exhibited a stronger negative charge value (−40 to −60 ζ), and the isoelectric point was confirmed to be 2.0. The strong negative charge of PAA-POSS-APBA@insulin particles at pH 7.4, which mimics the human body environment, indicates that they were stably dispersed. Because of its stable negative charge, boronic acid selectively binds with glucose to enable a rapid exchange reaction. The zeta-size analysis revealed that under acidic conditions, the PAA-POSS-APBA@Insulin particles exhibited significant swelling behavior, resulting in a broad size distribution. Specifically, the particle size increased to approximately 3.3 μm at pH 2, while it decreased to around 0.8 μm at pH 4, indicating a marked variation in particle size depending on the pH. However, in weakly acidic, neutral, and basic environments, e.g., pH of ≥6, the particles were confirmed to be uniform, with no size deviation and an average size of 0.4 μm. This behavior is consistent with previous findings [23], in which the boronic acid moiety of APBA formed a stabilized environment through interactions with PEG-modified insulin or glucose, maintaining a stable particle distribution with zeta potentials exceeding ±30 mV.

The insulin entrapment efficiency (EE) of the prepared PAA-POSS-APBA@insulin was 75.6% owing to the highly hydrophobic nature of POSS, which resulted in strong bonding with insulin. The loading capacity of PAA-POSS-APBA@insulin was 51.0%. This high LC is attributed to the synergistic effect of the 3D stereostructure of POSS and the electrostatic/covalent interactions between the boronic acid groups of APBA and the amino/hydroxyl groups of insulin.

**Figure 3:**
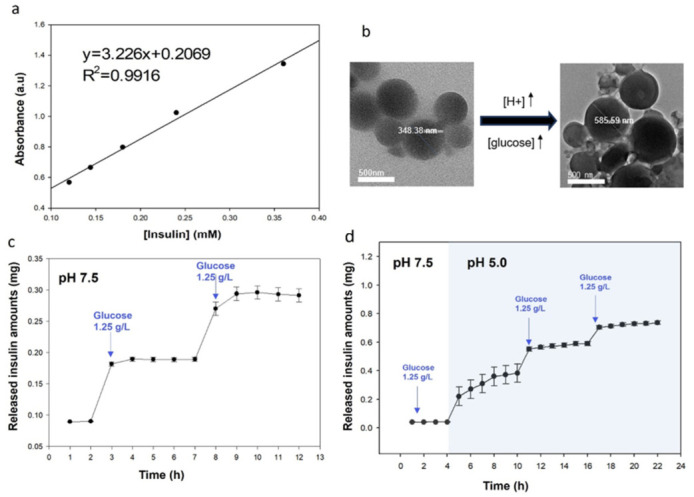
(**a**) Calibration curve of insulin. (**b**) TEM images of PAA-POSS-APBA@insulin with glucose added and particle changes under acidic conditions of pH 5.0. Arrows indicate increasing H^+^ and glucose concentration. In vitro glucose-responsive release of insulin from PAA-POSS-APBA@insulin NPs. Continuous insulin release of (**c**) POSS-APBA@insulin at pH 7.5 and (**d**) PAA-POSS-APBA@insulin from pH 7.5 to pH 5.0. Data are presented as mean ± standard deviation (*n* = 3).

The amount of drug released was calculated using an insulin calibration curve (Figure 3a). The size of PAA-POSS-APBA@insulin particles increased significantly to an average of 581 ± 74 nm when the glucose concentration increased under the acidic condition. This is because the carboxyl group of PAA is deprotonated and hydrophilic under neutral or alkaline conditions but is hydrophobic in an acidic environment; therefore, the main chain is expanded by the repulsion force, and the exchange reaction between insulin and sugar begins in the space created [34].

To clarify the release mechanism, it is essential to consider our previous findings where POSS-APBA alone exhibited high glucose sensitivity even at neutral pH [23], and PAA-POSS demonstrated superior pH-responsiveness compared to bulk PAA [24]. In this dual-responsive system, PAA-POSS-APBA@insulin did not release insulin at 125 mg/dL glucose in phosphate-buffered saline (PBS) at pH 7.4 but released insulin in a steep linear manner over time at pH 5.0. This suggests that at neutral pH, the PAA shell acts as a physical barrier that restricts glucose access to the internal APBA–insulin complexes. However, under acidic conditions, the PAA backbone was expanded due to the repulsive forces following conformational changes. This structural expansion allows glucose to penetrate the core and trigger the boronate-diol exchange reaction, displacing the bound insulin. When 125 mg/dL of glucose was added every 3 h while maintaining acidic conditions, insulin was rapidly released at the initial stage. This confirmed that in contrast to the release of POSS-APBA@insulin under neutral conditions, the PAA backbone was uncoiled under acidic conditions, providing an environment for the selective reaction between insulin bound to the boronic acid inside and sugar.

**Figure 4:**
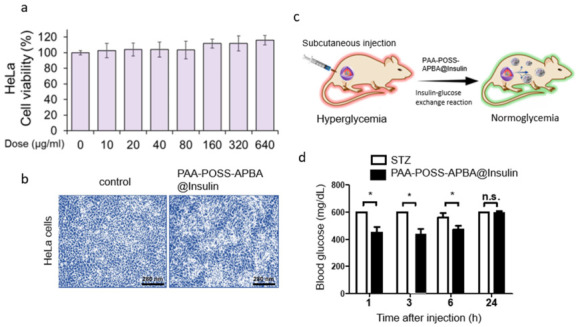
(**a**) Viability of HeLa cells treated with various concentrations of PAA-POSS-APBA@insulin for 24 h. (**b**) Morphologies of normal HeLa cells and cells treated with 640 µg/mL PAA-POSS-APBA@insulin after 24 h. (**c**) PAA-POSS-APBA@insulin delivery for T1D treatment using the STZ-induced diabetic mouse model. (**d**) Monitoring BG levels in vivo. PBS and BG levels in diabetic mice after subcutaneous injection of PAA-POSS-APBA@insulin. Data are presented as mean ± standard deviation (*n* = 3). Statistical significance was analyzed using two-way ANOVA. * *p* < 0.05 vs. Control; n.s., not significant.

Assessment of the toxicity of drug delivery systems when administered to the body is essential. As shown in Figure 4, HeLa cells derived from cervical cancer cells were used as primary cells to confirm cell growth and death, and a cytotoxicity test for PAA-POSS-APBA@insulin NPs was performed. Figure 4a shows that PAA-POSS-APBA@insulin NPs exhibited no HeLa cell toxicity up to a concentration of 640 μg/mL, and it can be confirmed that cell growth increased linearly with increasing concentration, indicating cytocompatibility. Figure 4b shows the morphologies of cells in the untreated group and after treatment with PAA-POSS-APBA@insulin NPs at a concentration of 640 μg/mL for 24 h. In contrast to the untreated group, the cytoplasm was developed and exhibited a relatively high-density morphology after treatment. These results suggest that PAA-POSS-APBA@insulin NPs have high biocompatibility and are applicable for drug delivery in the body. Regarding the physiological fate, the PAA shell ionizes and dissociates at pH 7.4, breaking the system into individual chains [35] and POSS components. As reported by Kumar et al. [36], these small hybrid fragments (typically <5.5 nm) are eliminated through renal clearance (urine), while any remaining silica–silicon moieties are cleared via the hepatobiliary pathway (feces). This dual-clearance mechanism ensures the carrier is safely removed from the body without long-term accumulation.

To evaluate the efficacy of insulin-loaded nanocomposites for treating hyperglycemia in T1D mice, PBS and PAA-POSS-APBA@insulin were injected subcutaneously into STZ-induced diabetic mice. PBS or untreated healthy mice were used as controls. The BG levels of treated mice were monitored using a glucometer at predetermined times. As shown in Figure 4d, the average BG levels of T1D mice treated with PAA-POSS-APBA@insulin (4.0 IU/kg) for >6 h were lower than those of the PBS-treated group, and the BG levels were maintained stably with an average of 470 mg dL^−1^. We attribute this remarkably long-lasting effect to sustained and smart glucose-responsive insulin release. According to the paper, in the case of rats treated with free insulin, BG levels rapidly decreased to the hypoglycemic range (~50 mg dL^−1^) within 1 h and recovered to the hyperglycemic range (~400 mg dL^−1^) within 3 h, owing to the rapid initial diffusion of free insulin in vivo [37,38,39,40,41,42]. Compared with other studies [43,44] where the treatment concentration was low, normal BG can be managed by adjusting the treatment concentration of PAA-POSS-APBA@insulin. We confirmed that PAA-POSS-APBA@insulin maintained BG homeostasis in the body for at least 6 h. We have added a quantitative comparison table (Table 1) to clearly benchmark our system against recent glucose-responsive delivery systems.

## 4. Conclusions

Dual-responsive glucose and pH smart carriers based on PAA-POSS-APBA were prepared and evaluated for insulin delivery. Specifically, PAA-POSS-APBA@insulin (386 ± 69 nm, EE: 75.6%, LC: 51.0%), a spherical drug delivery carrier enclosing insulin, was synthesized using POSS as a hydrophobic material and PAA and APBA, which respond to pH and glucose, respectively. This drug carrier has dual reactivity with pH and glucose, and its synthesis was confirmed through FT-IR spectroscopy, which verified that the particles were stable at different pH values through the zeta-potential data. In particular, PAA-POSS-APBA@insulin exhibited highly sensitive drug delivery properties, in which the backbone of PAA was expanded under acidic conditions, and insulin bound to boronic acid could rapidly and selectively react with trace amounts of d-glucose. PAA-POSS-APBA@insulin NPs exhibited no HeLa cell toxicity up to a high concentration of 640 μg/mL, and the cell growth rate increased with the concentration, indicating biocompatibility. The average blood sugar level of diabetic mice treated with POSS-APBA@insulin (4.0 IU/kg) was reduced for >6 h, and the blood sugar was stably maintained, suggesting that this hybrid material using POSS has potential applications in self-regulated insulin delivery, actuators, and separation systems with sensitivity to glucose.

## Figures and Tables

**Table 1:** Comparison of glucose-responsive insulin delivery systems for in vivo blood glucose regulation.

System	Mechanism	Size	LC (%)	EE (%)	In Vivo Glucose Control	Duration (h)	Key Limitation	Ref.
Gox enzyme-based hyaluronic acid	Glucose	118 nm	8.7	60–80	Rapid decrease	~6	Enzyme instability and ROS	[45]
d-GRPs Peptide based microneedle	H_2_O_2_/hypoxia	~150 nm	3.2		Stable regulation	6~8	Complex system	[46]
poly(N-isopropylacrylamide P(NIPAAm)-PBA Hydrogel	Glucose	15mm dim. Tube-gel			Glucose-dependent release	~5 ^1^	Slow response	[47]
Chitosan-enzyme	pH or glucose	256 ± 18 μm	45		Partial control	~10	Weak dual response	[48]
PAA–POSS-APBA	pH + glucose dual-responsive	386 ± 69 nm		75.6	Sustained glucose reduction	>6	None	This work

^1^ In a thermostated flow chamber connected with an inflow circuit equipped with a valve alternately switching the flow with high and low glucose concentrations.

## Data Availability

The original contributions presented in the study are included in the article/Supplementary Materials, further inquiries can be directed to the corresponding author.

## Supplementary Materials

The following supporting information can be downloaded at: https://www.mdpi.com/article/10.3390/polym18101209/s1. Figure S1: TEM images of PAA-POSS-APBA@Insulin with various scales; Figure S2: TGA thermogram of PAA-POSS-APBA@Inuslin.

## Abbreviations

^1^H-NMR	Proton Nuclear Magnetic Resonance
APBA	3-Aminophenylboronic Acid
ATCC	American Type Culture Collection
BG	Blood Glucose
CAGE	Choline-Based Ionic Liquid
CD	Circular Dichroism
DM_ave_	Average Diameter
Da	Dalton
DMEM	Dulbecco’s Modified Eagle Medium
DMSO	Dimethyl Sulfoxide
EE	Entrapment Efficiency
FBS	Fetal Bovine Serum
FE-SEM	Field-Emission Scanning Electron Microscopy
FT-IR	Fourier Transform Infrared Spectroscopy
GOx	Glucose Oxidase
HCl	Hydrochloric Acid
HDF	Human Dermal Fibroblast
HeLa	Human Cervical Cancer Cell Line
HUVE	Human Umbilical Vein Endothelial
IRB	Institutional Review Board
IU	International Unit
KBr	Potassium Bromide
LC	Loading Capacity
MTT	3-(4,5-Dimethylthiazol-2-yl)-2,5-Diphenyltetrazolium Bromide
MW	Molecular Weight
MWCO	Molecular Weight Cutoff
NaOH	Sodium Hydroxide
NPs	Nanoparticles
PAA	Poly(acrylic acid)
PBS	Phosphate Buffered Saline
PBA	Phenylboronic Acid
PEG	Polyethylene Glycol
POSS	Polyhedral Oligomeric Silsesquioxane
SEM	Scanning Electron Microscopy
STZ	Streptozotocin
TEM	Transmission Electron Microscopy
TGA	Thermogravimetric Analysis
THF	Tetrahydrofuran
UV–vis	Ultraviolet–visible
ζ (Zeta)	Zeta potential
