# Supplementary material for: pH and Glucose Dual-Responsive Hybrid Polymeric Smart Insulin Carrier for Diabetes Treatment
Source: Polymers (Basel). 2026 May 15;18(10):1209. doi: 10.3390/polym18101209 (PMC13211201; doi:10.3390/polym18101209)
Supplement: Supplementary file 1 [file polymers-18-01209-s001.zip › polymers-4196995-supplementary.pdf]

Supporting Information

pH and Glucose Dual-Responsive Hybrid Polymeric Smart Insulin Carrier for Diabetes Treatment

Kyu Oh Kim

Department of Fiber System Engineering, Dankook University, 152, Jookjeon-ro, Suji-gu, Yongin-si, Gyeonggi-do, 448-701, Republic of Korea

* Correspondence: kokim95@dankook.ac.kr (K.O. Kim)

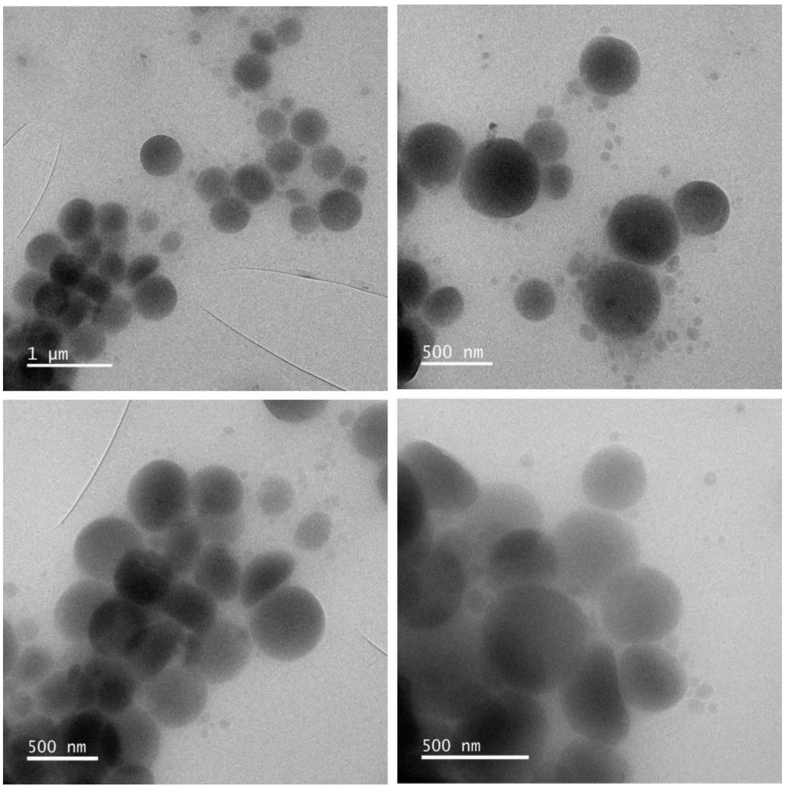

Figure S1. TEM images of PAA-POSS-APBA@Insulin with various scales

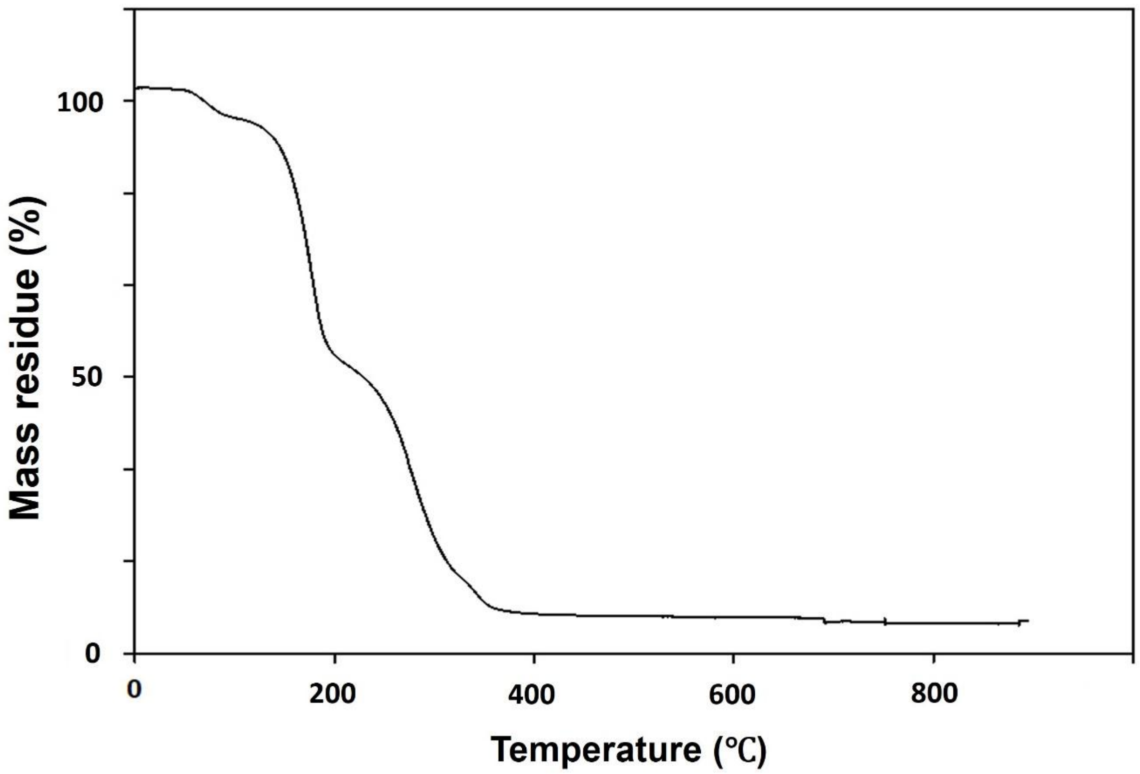

Figure S2. TGA thermogram of PAA-POSS-APBA@Inuslin.
